# On the Use of Transfer Entropy to Investigate the Time Horizon of Causal Influences between Signals

**DOI:** 10.3390/e20090627

**Published:** 2018-08-22

**Authors:** Andrea Murari, Michele Lungaroni, Emmanuele Peluso, Pasquale Gaudio, Ernesto Lerche, Luca Garzotti, Michela Gelfusa, JET Contributors

**Affiliations:** 1 Consorzio RFX (CNR, ENEA, INFN, Universita’ di Padova, Acciaierie Venete SpA), I-35127 Padova, Italy; 2 Associazione EUROfusion—University of Rome “Tor Vergata”, Via Orazio Raimondo, 18, 00173 Roma, Italy; 3 EUROfusion Programme Management Unit, JET, Culham Science Centre, Abingdon OX14 3DB, UK; 4 LPP-ERM/KMS, Association EUROFUSION-Belgian State, TEC partner, Brussels 1000, Belgium

**Keywords:** transfer entropy, mutual information, Pearson correlation coefficient, time series, causality detection, sawteeth, pacing, ELMs, pellets

## Abstract

Understanding the details of the correlation between time series is an essential step on the route to assessing the causal relation between systems. Traditional statistical indicators, such as the Pearson correlation coefficient and the mutual information, have some significant limitations. More recently, transfer entropy has been proposed as a powerful tool to understand the flow of information between signals. In this paper, the comparative advantages of transfer entropy, for determining the time horizon of causal influence, are illustrated with the help of synthetic data. The technique has been specifically revised for the analysis of synchronization experiments. The investigation of experimental data from thermonuclear plasma diagnostics proves the potential and limitations of the developed approach.

## 1. Detecting Causal Influence between Time Series

In many fields of science, the causal relations between systems have to be investigated. Very often, the information about the phenomena, whose causal mutual influence is to be studied, is available in the form of time series. Of course, a general definition of causality is not available. On the other hand, in recent years, the concept of information flow has been used as an indicator of causal influence between systems [1]. In this framework, the phenomena under study are represented by stochastic variables and the information flow between them is quantified with tools, such as the Pearson correlation coefficient (Pcc) and the mutual information (MI). More recently a quite powerful tool, transfer entropy (TE), has been introduced [2] and its use is spreading quite fast, complementing or superseding alternative approaches such as Granger causality (GC) [3].

In this paper, the advantages and limitations of transfer entropy, in the analysis of the coupling between time series, are illustrated with some numerical examples. Then the issue of determining the time horizon in synchronization experiments is addressed. In particular, the coupling in pacing experiments is investigated with specific attention to real life examples from high temperature plasma physics.

Our human environment is full of oscillating systems, both natural and man-made. Electromagnetic waves, circulation systems in mammals, and some chemical reactions all proceed at a periodic or quasi-periodic, oscillating pace. Most of these systems are not isolated and, sometimes, even minor interactions can affect their rhythms and bring them in synchrony. Recently, synchronization by external forcing has attracted a lot of attention as a method to influence delicate systems. In some cases, the best way to control the dynamics is some sort of soft interaction, to bring the target system in synchrony with some external oscillation. The control of chaotic systems is a good example. 

In some areas, such as thermonuclear fusion, the assessment of the actual effectiveness of an external perturbation is quite complex. Poor accessibility for measurements is a typical issue. Additionally, in some cases, there is a need to influence quasiperiodic systems whose behaviour needs to be only slightly changed. The degree of influence actually exerted by the external driver can therefore be quite delicate to assess. In some fields, physics models of the dynamics are not available and, therefore, the quantification of the driver efficiency can be performed only in a statistical way, over many examples on not on an individual basis. In particular, it can become essential to determine the “time horizon” of the causal influence, within which the driving system actually exerts an effect on the target system. This aspect is crucial to quantify the performance of the driving schemes, because synchronization beyond that time horizon has to be considered the result of random coincidences (see Section 4). In this perspective, the power of TE can be essential.

The paper is structured as follows. The definition of transfer entropy is introduced in the next section, together with the Pearson correlation coefficient and the mutual information. The added value of transfer entropy and some of its deficiencies are illustrated with the help of numerical examples in Section 3, focussing on the determination of the causal influence time horizon. Examples of the application to synchronization experiments in tokamaks are presented in Section 4. Discussion and future developments are the subject of the last section. 

## 2. Pearson Correlation Coefficient, Mutual Information, and Transfer Entropy 

This section provides and introduction to the concept of transfer entropy, in the context of the other main statistical indicators, normally deployed to investigate the correlation between time series. The Pearson coefficient *ρ* is an indicator typically used to quantify the linear relation between two stochastic variables, *X* and *Y*. The mathematical definition of the Pearson coefficient *ρ* is:

(1) $$\;\rho_{X,Y\;}=\frac{E\left[\left(X-\mu_{X}\right)\left(Y-\mu_{Y}\right)\right]}{\sigma_{X}\sigma_{Y}}$$

where *µ_X_* is the mean of the variable *X*, *µ_Y_* is the mean of the variable *Y*, *σ_X_* is the standard deviation of *X* and *σ_Y_* is the standard deviation of *Y*. The correlation coefficient can range in value between −1 and +1 and reveals two aspects about the linear relation between variables: its strength and direction. The direction is determined by the sign, which indicates whether the variables are positive correlated or anti-correlated. With the appropriate reservations, the sign of the Pearson coefficient *ρ* can be interpreted as the direction of causal influence.

The main limitation of the Pearson coefficient is its linear character. A traditional alternative quantifier, to take into consideration also the nonlinear influence between variables, is the mutual information. Intuitively, the mutual information *I(X*;*Y)* measures the quantity of information that the two variables *X* and *Y* have in common. Probably the best starting point, to understand the meaning of the mutual information and then of the transfer entropy, is the Kullback–Leibler Divergence (*D_KL_*). The *D_K_*_L_ is an integral, which quantifies the difference between two probability distribution functions *p(x)* and *q(x)*. The *D_KL_* is defined as:

(2) $$\;D_{KL\;}\left(P||Q\right)=\int p\left(x\right)\cdot \ln\left(\frac{p\left(x\right)}{q\left(x\right)}\right)dx$$

The Kullback–Leibler Divergence assumes positive values and is zero only when the two probability distribution functions (pdfs), *p* and *q*, are exactly the same. Therefore, the smaller the *D_KL_* is, the more similar the two pdfs are. In our application, *p* and *q* are the pdfs of the time series *X* and *Y*.

The mutual information is meant to quantify the information that two random variables *Y* and *X* have in common and it can be expressed in a form interpretable in terms of the *D_KL_*. Indeed, a typical definition of the mutual information is:

(3) $$\;I\left(X;Y\;\right)=\underset{y\in Y}{\sum }\underset{x\in X}{\sum }p\left(x,y\right)\log\left(\frac{p\left(x,y\right)}{p\left(x\right)p\left(y\right)}\right)$$

where *p(x,y)* is the joint probability function of *X* and *Y*, *p*(*x*) and *p*(*y*) are the marginal probability distribution functions of *X* and *Y,* respectively. Thus, the higher the mutual information, the more different their joint pdf is from the case of independence, when the joint pdf factorizes into the product of the two individual pdfs. MI includes nonlinear interactions but is affected by the obvious limitation that it cannot provide any information about the directionality of the causal relation. Moreover, the MI does not take into account the memory effects of the signals (see the following).

Almost two decades ago, the notion of transfer entropy was formalised, to provide a new and more powerful tool to investigate causal effects [2]. The main motivation, behind the development of the TE formalism, resides in the observation that the lagged mutual information *I(Y_t_;X_t_*_−*s*_*)* is not adequate to properly quantify the strength of the causality between the time series *X* and *Y*. The lagged mutual information is unsatisfactory because it does not take into account the target history. Basically *I(Y_t_*;*X_t_*_−*s*_*)* cannot discriminate the actual mutual causal effect, between the signals of interest, from the memory of the system *Y* on itself. Transfer entropy has been specifically developed to take into account the effect of the history of the signals. In this context, its most useful definition is the following:

(4) $$\;T_{X\to Y\;}=\underset{y_{t+1},y_{t}^{n},x_{t}^{m}}{\sum }p\left(y_{t+1},y_{t}^{n},x_{t}^{m}\right)\log\frac{p\left(y_{t+1}|y_{t}^{n},x_{t}^{m}\right)}{p\left(y_{t+1}|y_{t}^{n}\right)}$$

In Equation (4), $y_{t}^{n}\;$= (*y_t_, y_t_*_−1_*, …, y_t_*_−*n*+1_) and $x_{t}^{m}$= (*x_t_, x_t_*_−1_, *…*, *x_t_*_−*m+*1_) are the *n* and *m* orders of the Markov processes X and *Y*. TE can, therefore, be interpreted as the degree of uncertainty in the present *Y* given the past state of *Y* and *X*, in excess of the degree of uncertainties resolved by *Y*’s own past. By taking into account the past of the signals, TE also provides clear information about the direction of the causal interaction. With regard to the numerical calculations, MI and TE have been computed with the method of the differential entropy described in detail in [4].

It has to be mentioned that recently significant limitations of TE, to properly quantify the information transfer in complex cases, have been pointed out. Causal influences can be either underestimated or overestimated by TE, depending on the situation, when the relationship between the systems is not dyadic, but polyadic. As discussed in more detail in the last section, these issues, important as they are, do not raise particularly concerns for the type of studies reported in this paper. 

## 3. Assessing Causality: Numerical Tests

In this section, the competitive advantages of TE with respect to the Pearson correlation coefficient and the mutual information are illustrated with the help of numerical examples. The main rationale behind the choice of the synthetic data reported in this section is two-fold. First, the numerical data investigated are at least of the same level of complexity than the experimental signals analysed in Section 4. Therefore, together with numerical simulations based on first principles (see Section 4), these examples support the conclusions reached in the analysis of experiments. Secondly, the use of numerical signals helps clarifying the properties of the various indicators in controlled conditions. 

In support to the investigation of sawteeth pacing with Ion Cyclotron Resonance Heating (ICRH) modulation (see Section 4), the properties of TE and the other indicators are exemplified by the analysis of the two following equations:

(5) $$\;f_{1}(t)\;=5\sin\left(5t\right)+t-0.1t^{2}+\mathrm{noise}$$

(6) $$\;f_{2}\left(f_{1},t\;\right)=10\sin\left(t\right)-cc\left(t-\tau \right)+cc|f_{1}\left(t-\tau \right){|}^{0.5}$$

**Figure 1:**
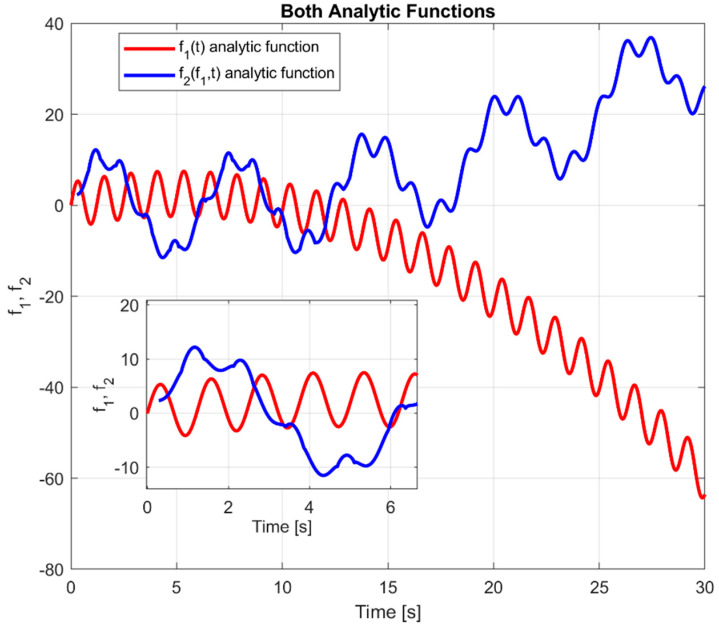
Graphical representation of the analytic expressions *f*_1_ and *f*_2_. In the insert, the first *f*_2_ period is expanded for clarity sake.

*f*_1_ is the independent system exerting a causal influence on the system *f*_2_. The coupling coefficient *cc* is set at a constant value of 0.5 for the numerical studies reported in the rest of this section. The time delay *τ* is fixed at a value of 8 in the following analysis; this is to be interpreted as the causality time horizon introduced in the first section. Of course, it has been checked that the results do not change qualitatively for different values of these two parameters, coupling coefficient and time lag. The two Equations (5) and (6) are represented graphically in Figure 1. The time axis covers a period from 0 to 30 in intervals of 0.02, for a total of 1500 steps. It is worth mentioning that the complexity of the reported numerical example is at least of the same level than the experimental cases of sawteeth pacing with ICRH modulation discussed later in the paper; for example, contrary to the real data discussed in Section 4, the synthetic data are not globally stationary.

Additionally, to simulate realistic experimental conditions, in terms of uncertainties in the signals, a noise of Gaussian pdf and standard deviation of 3 has been added to the function *f*_1_. Moreover, since the proposed analysis is statistical in nature, 21 different realisations of the functions *f*_1_ and *f*_2_ have been generated. 

**Figure 2:**
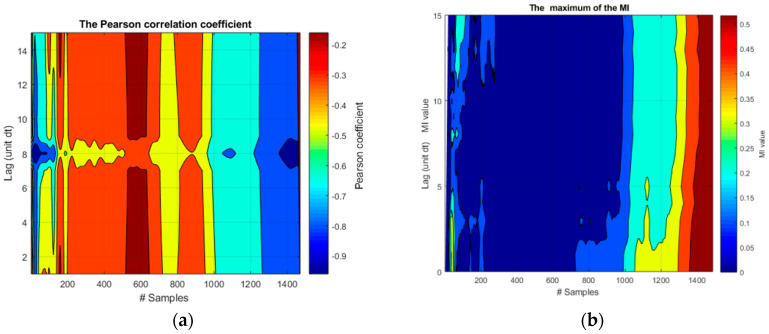
Application of the Pearson correlation coefficient and the Mutual Information to the 21 realizations of the functions *f*_1_ and *f*_2_. (**a**) Pearson correlation coefficient; (**b**) Mutual information. On the abscissa axis, the label “#Samples” is meant to indicate the time steps for the synthetic signals.

The application of the Pearson correlation coefficient and the Mutual Information to the 21 realizations of functions *f*_1_ and *f*_2_ is reported in Figure 2.

As can be easily understood, from the simple inspection of the plots in this figure, neither indicator can determine the right time horizon of the causal influence between the two signals. The Pearson correlation coefficient always finds a negative correlation between the two signals, which changes dramatically with time. As expected, this indicator cannot detect the fact that the negative correlation is in reality due to a constant nonlinear term. Moreover, the Pearson correlation coefficient provides no robust estimate of the proper lag time. MI, in its turn, increases toward the end of the time series but there is no relation with the lag time. This is due to the confusing effects of the common trend and the fast oscillations. 

**Figure 3:**
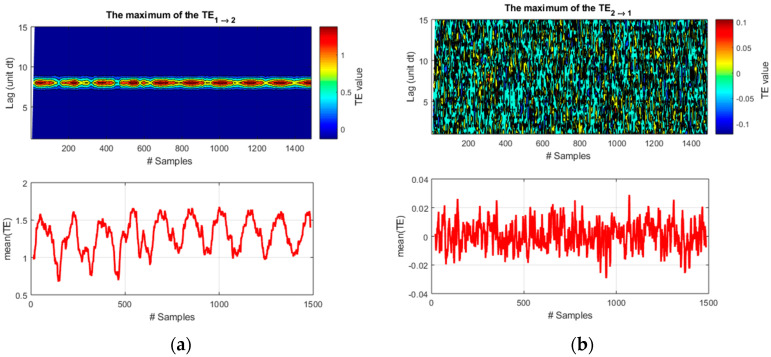
(**a**) Representation of transfer entropy (TE) for causality from *f*_1_ to *f*_2_; (**b**) Representation of TE for causality from *f*_2_ to *f*_1_. The correct lag time of 8 time units is detected only for the right causal influence *f*_1_ to *f*_2_. On the abscissa axis, the label “#Samples” is meant to indicate the time steps for these synthetic signals.

The transfer entropy fares much better. The plots of Figure 3 show quite clearly that TE manages to identify the direction of the causal influence from *f*_1_ to *f*_2_. Moreover, for causality from *f*_1_ to *f*_2_, TE presents clearly distinct peaks for the correct lag time of 8 time units.

**Figure 4:**
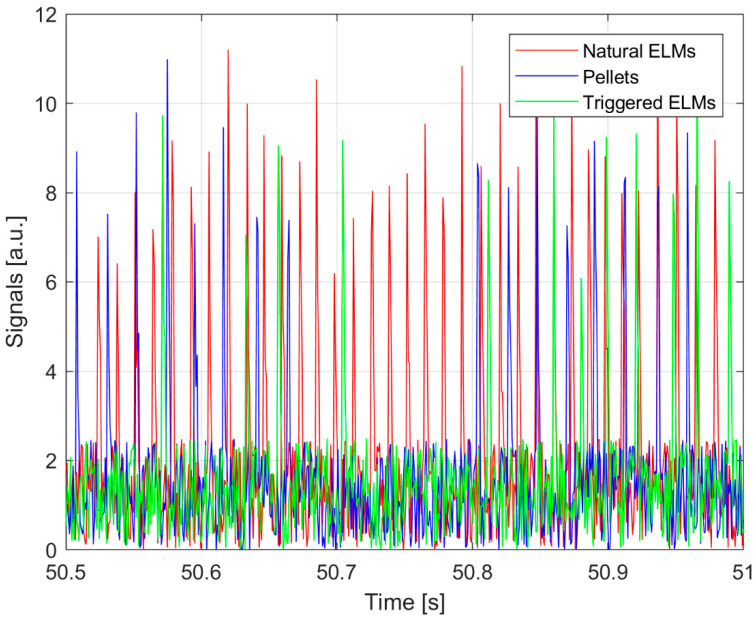
Graphical representation of the simulated synthetic spiky signals generated. Red: the unperturbed target signal. Green: the target signals plus the effects of the driver (see text for detailed explanation).

As mentioned, a second specific example is shown to support the applicability of TE to the analysis of the causal influence between spiky signals. This is motivated by the fact that the signature of various instabilities can consist of quite abrupt variations in the signals. It is therefore necessary to double-check that TE can properly handle even this typology of data, which are characteristics of the edge localized modes (ELMs) discussed in Section 4. An example of the synthetic data simulated is given in Figure 4. Triangularly-shaped pulses, with slightly different slopes and heights, have been generated first to simulate the target system (red spikes in Figure 4). Two trains of triangularly-shaped pulses represent the driver (blue triangles in Figure 4). Then, for 60% of the driver spikes, a corresponding spike has been generated and summed to the unperturbed train of spikes (green triangles in Figure 4). For the case of the first train, the delay of the generated spikes in the target system is 20 samples with a variability of ±3 samples, uniformly distributed; for the case of the second train, the delay of the generated spikes in the target system is 10 samples ±3 samples, again uniformly distributed. As usual Gaussian noise has been added to all the time series to obtain a signal to noise ratio of about ten. To simulate a realistic behaviour, as the one reported in Section 4.2, the blue spikes are to be considered the driver and the union of the red and green signals compose the target. The results reported in the rest of this section have been obtained by averaging 21 couples of time sequences. Again, this numerical case is of the same order of complexity as the experimental ones investigated in the next section.

**Figure 5:**
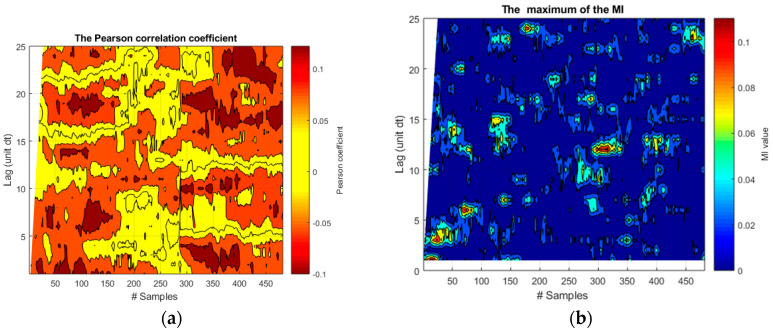
(**a**) Pearson correlation coefficient for the 21 signal couples one of which is shown in Figure 4; (**b**) Mutual Information for the 21 signal couples. A sliding window of 60 samples has been implemented.

**Figure 6:**
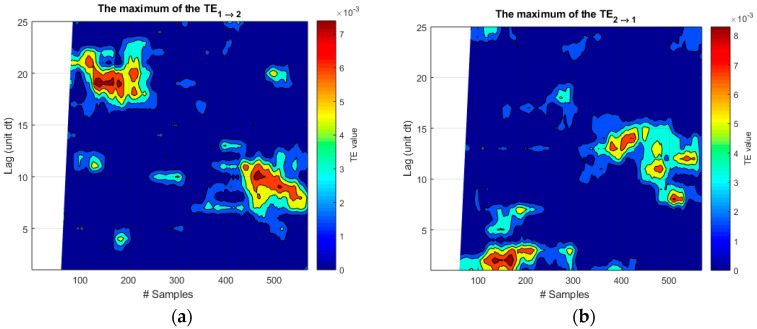
Transfer entropy for the 21 signal couples, one of which is shown in Figure 4. (**a**) TE in the right causal direction. The two different delays of the two trains of driver signals are clearly identified; (**b**) TE in the wrong causal direction. In this case the lag times are not identified correctly; more importantly, the values of TE are comparable to the ones of the left plot and, therefore, the direction of causality is not properly identified.

Again, also for this typology of data, the Pcc and MI have great difficulties to provide any meaningful indications about the time lag, as shown in Figure 5. In the case of Pcc, the correlation coefficient presents very sparse regions of similar amplitudes, which are almost impossible to interpret. The MI is always very close to zero with some small exceptions, which have no relation with the actual coupling between the systems. It is also to be noted that these unsatisfactory outputs have been obtained even implementing a quiet optimized sliding windows of 60 time steps. On the other hand, a direct application of TE, without any fine tuning, identifies very clearly the two intervals of coupling with the right lags of the causal influence, as illustrated in the left plot of Figure 6. On the other hand, the directionality of the causal influence is not properly identified even by TE for these spiky signals. This fact can be seen by comparing the two plots of Figure 6, which show very similar levels of TE in both directions. This aspect is not relevant for the experiments studied in Section 4, whose causal directionality is clear since they are based on external perturbations. On the other hand, this analysis reveals a limitation of TE in handling spiky signals, which is reported for the first time in this paper.

The lag time of the reported synthetic cases is the essential quantity to determine because it is meant to simulate the causality time horizon in the experimental signals. Formally, the causality time horizon can be defined as the interval within which there is 95% of probability that the driver actually triggered the desired response in the target system. How the causality horizon is calculated in the case of the experiments is described in detail in the next section, together with a discussion about the related uncertainties. It is also worth emphasising that the examples reported are representative of the general behaviour of the three indicators. It has indeed been confirmed by a series of numerical tests, with different synthetic data, that transfer entropy always outperforms the Pearson correlation coefficient and the mutual information, if the causal relations between the function includes nonlinear terms and memory or seasonal effects. These effects are the main ones at play in the actual experiments, whose signals are analysed in the next section. More involved situations and additional complications can compromise the reliability of TE but, as discussed in the conclusions, such considerations are not relevant for the studies reported in this paper.

## 4. Determining the Causality Horizon: Pacing Experiments in Thermonuclear Fusion

Control of instabilities, such as sawteeth and ELMs, is considered an important ingredient in the development of fusion reactor-relevant scenarios. Since their effects are complex and not necessarily all negatives, these instabilities have to be properly managed more than completely eliminated. The experiments, to prove their synchronization with external pacing systems and the issues related to the interpretation of their results, are discussed in detail in the next two subsections.

### 4.1. ICRH Modulation for Sawteeth Pacing 

The Joint European Torus (JET), the most reactor-relevant tokamak in operation in the world, presents relaxation oscillations in the centre of the plasma. These relaxations manifest themselves as non-linear oscillations, which look like a sequence of sawteeth, from which they are named. Indeed, sawteeth follow a cycle with a slow increase in the plasma temperature (and other parameters) in the plasma centre, followed by an abrupt crash, which usually brings the plasma back to conditions very similar to the ones at the start of the oscillating cycle. As an effect of the sawtooth crash, an appreciable fraction of energy is ejected from the plasma core into the external regions, where it induces an increase in the temperature. The radial position, at the boundary between the regions of decrease and increase of the temperature, is called the sawtooth inversion radius [5,6].

If their crash is not too large, sawteeth, first reported in [7], do not affect plasma performance to an unacceptable level. They can induce a moderate reduction in confinement but, in the reactor, they could have the beneficial effect of expelling impurities and helium ash from the plasma core. On the other hand, if the sawteeth are too large, they can trigger more deleterious instabilities, such as neo-classical tearing modes (NTM), which degrade plasma confinement and can even induce disruptions. Control of the sawteeth period is, therefore, extremely important to control impurities, preserve confinement, and avoid premature terminations of the discharges. 

Different methods have been investigated to control the period of sawteeth in tokamaks. One of them is based on the modulation of the ICRH power. Many types of electromagnetic waves can propagate in magnetised plasmas. Among the phenomena involving them, the most important is probably resonant absorption, which can be used for heating by non-collisional mechanisms [8]. ICRH can be used to transfer energy directly to the minority ions and, in addition to pure heating, in JET it has often been deployed to investigate the physics of fast particles [9]. More recently, and mainly since the installation of the ITER-like wall (ILW), ICRH has contributed significantly to impurity control in the framework of scenarios development [10]. In this perspective of avoiding dangerous NTMs and reducing the adverse effects of heavy impurities, ICRH control of the sawtooth cycle is very promising. Different methods for sawtooth pacing with ICRH have been recently tested in JET-ILW [10,11]. ICRH is believed to act on the pressure and distribution function of energetic ions in the plasma, which have a stabilizing effect on sawteeth. This paper reports on experiments based on using central ICRH power, which stabilises the sawteeth, resulting in longer periods; then the crash is induced by briefly switching the ICRH power off. From a plasma physics perspective, the time delay, between the notch in the ICRH power and the sawtooth crash, is expected to be of the order of the ions slowing-down time, which can be estimated theoretically. Therefore, the interval, between the notch in the ICRH power and the time of the sawtooth crash, is the fundamental quantity to interpret the physical mechanism responsible for the coupling.

**Figure 7:**
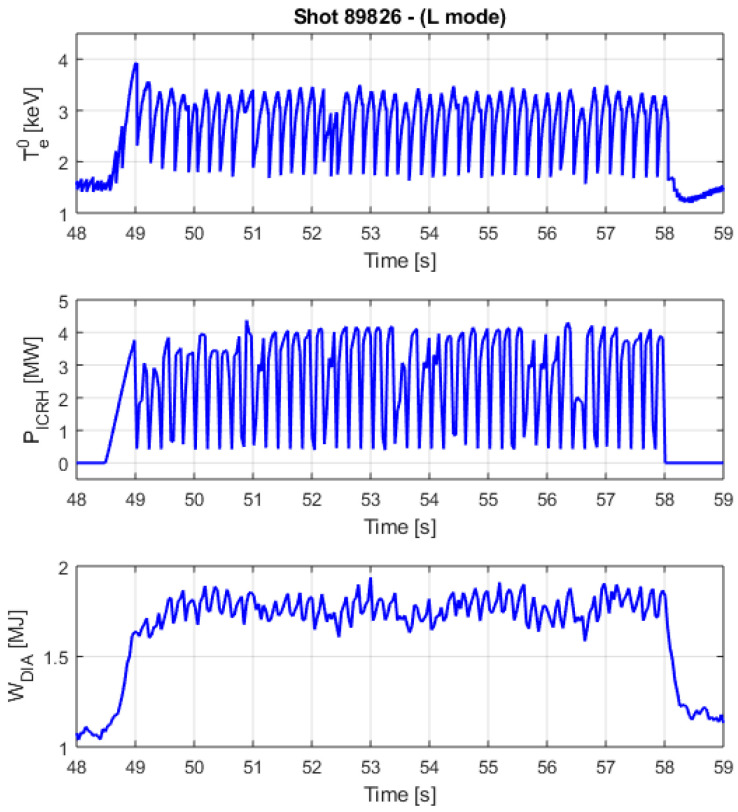
An example of sawteeth pacing with 5 Hz ICRH modulation for a JET ILW L mode discharge. The ICRH is modulated (150 ms on and 50 ms off). The maximum power is 4 MW in a minority heating scheme with 4% of H in D. From top to bottom: central electron temperature, ICRH power and plasma internal energy.

Irrespective of the technical details and the physical mechanism stabilizing the sawteeth, a typical difficulty of these experiments is their interpretation. Indeed, to determine the efficiency of the pacing, the number of sawteeth effectively triggered must be calculated reliably. This is not a simple task as can be appreciated from inspection of Figure 7.

The signal waveforms are quite complex and the detailed determination of exactly which sawteeth have been triggered by the undertaken action is a very delicate issue. Indeed, since sawteeth are quasiperiodic, if enough time is allowed to elapse, they would occur almost always after a notch in the RF. Therefore the occurrence of statistical coincidences cannot be ruled out “a priori”. Since no first principle physical model of the interaction between ICRH and sawteeth is available, it is impossible to determine the trigger efficiency on an individual basis. The statistical approach of the time horizon of causal influence has therefore been adopted. The time lag of maximum information transfer between the signals of the ICRH power and the sawteeth has been determined with the help of TE, as the time when TE reaches a maximum. The sawteeth occurring within this interval, with respect to the last notch, are considered triggered by the ICRH modulation. The ones, which occur with a delay longer than this lag time, are deemed to be due to the natural dynamics of the plasma and not to have any causal relation with the ICRH modulation. This analysis is performed individually for each discharge, to take into account the specific conditions of the plasma.

The results obtained with TE are summarised in Table 1 for a series of JET discharges both in L and H mode of confinement. In Table 1 the uncertainties in TE have been obtained by fitting a parabola to the maximum and determining the width at the 95% of that value. The uncertainties in the slowing down times are the spread in this value due the varying temperatures. For Pcc and MI the uncertainties have been calculated as the difference between their maximum and minimum values over the shot. 

The TE time of maximum causal influence, between the ICRH modulation and the sawteeth response, is in very good agreement with the slowing down time of the ions. The estimates obtained with the Pearson correlation coefficient and the mutual information are also reported in Table 1. Inspection of the table reveals that the results obtained with the Pcc and the MI are clearly of inferior quality. The MI very often gets the horizon of causal influence completely wrong. The Pearson correlation coefficient is a bit more accurate, but only because the uncertainties in its estimates are higher by almost an order of magnitude. The estimates obtained with Pearson correlation coefficient, therefore, cover such large ranges that often include the right value. As a consequence, the values provided by both the Pearson correlation coefficient and the MI would have very limited practical and theoretical use in these applications. 

### 4.2. ELM Pacing with Pellets 

The most important instabilities affecting the edge of H mode plasmas are the ELMs. They almost always affect significantly the energy confinement by degrading the edge transport barrier [8]. Since this degradation of the edge confinement is quite abrupt, energy and matter are expelled from the plasma on sub-millisecond time scales, which can cause unacceptable damage to the divertor in the next generation of devices and in the reactor. Various investigations of ELMs instabilities have produced contrasting results about their dynamics. Irrespective of the details about their physics, in the perspective of ITER and DEMO it is indispensable to control ELMs carefully, to alleviate their detrimental effects on the plasma facing components. Indeed, DEMO will probably have to be operated in ELM-free scenarios. On ITER, some active ELM control is also considered essential. Therefore, in the last years various forms of ELM pacing techniques have been tested. One of the most promising methods consists of pacing ELMs with small pellets of frozen deuterium [12,13]. The long term objective of this approach would consist of controlling the ELM frequency by triggering them with pellets. The ELM frequency is meant to be adjusted so that the gradients at the edge would not have time to increase excessively between subsequent ELMs. In this way, it is expected that the expulsion of energy and matter can be kept to manageable levels, not reaching values capable of damaging the plasma facing components. These pacing experiments are other typical cases of synchronization techniques.

**Figure 8:**
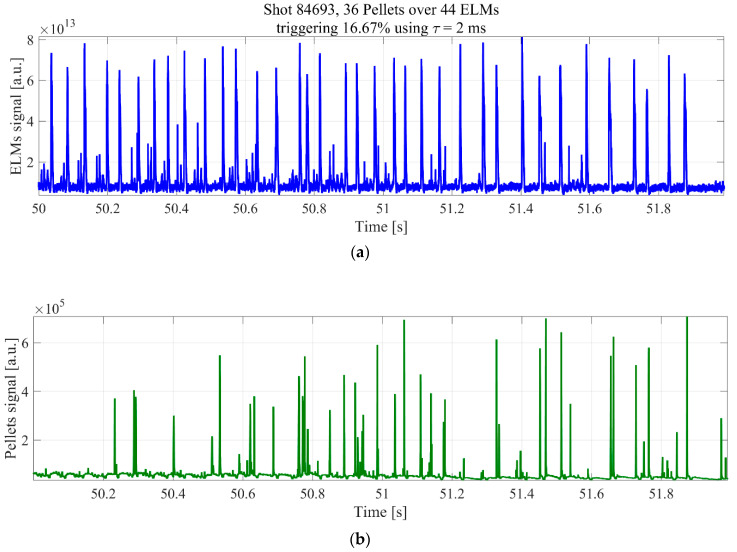
(**a**) *D_α_* signals identifying the occurrence of Edge Localized Modes (ELMs); (**b**) *D_α_* signal showing the time slices when the pellets enter the plasma.

Again one of the main difficulties in developing robust ELM pacing schemes resides in the interpretation of the experimental results, because these instabilities are also periodic or quasi periodic in nature. As a consequence, if enough time is allowed to elapse, an ELM always occurs after any sudden perturbation. To properly evaluate the triggering effectiveness of pellets, statistical indicators, to reliably determine the time interval during which pellets can really have a triggering capability, are indispensable. This means identifying the time horizon over which pellets can have a causal influence on the ELM dynamics. The challenge posed by this task can be appreciated by inspection of Figure 8, which reports the *D_α_* for ELMs and pellets in a JET discharge with the ITER-like wall (ILW). It is evident how deciding which pellets have triggered an ELM is quite problematic, particularly if one takes into account the non-negligible level of noise in the measurements. On JET, it has typically been assumed that a pellet can be effective in triggering an ELM only if the time elapsed between the two events is less or equal to 2 ms. In order to falsify this assumption, TE has been applied to a set of eight JET pulses, in a series of experiments explicitly meant to investigate various settings of the pellet pacing system: 82885, 82886, 82887, 82889, 84688, 84690, 84693, and 84696. The analysis relies on the *D_α_* emission to detect the occurrence of both the ELMs and the arrival time of the pellets in the plasma [12,13]. For these experiments, the detected signals are of adequate quality to permit reliable estimates.

For all discharges investigated, TE shows a slight increase for a short interval, reaches a quasi-constant level, and then decreases quite sharply for longer lag times. This is a behavior not unusual in many dynamical systems; the triggering event exhibits a noticeable influence for a specific interval, beyond which it becomes quickly ineffective. Pellets show a similar behavior, a fact that can be explained by remembering that ELMs are relaxation instabilities. Therefore, if pellets reach the plasma edge too far away from the completion of the natural ELM cycle, they have less chance to be effective. On the other hand, if pellets arrive to close to the end of the natural ELM cycle, they are less capable of destabilizing a quite stable plasma edge. The causality horizon, defined as the interval for successful triggering of the ELMs, can be calculated as the time point when the TE decays to 95% of its peak value. This choice of 95% interval has been made on the basis of many numerical tests, with pulses with shape and level of noise similar to the experimental values [14,15]. The causality horizon calculated in this way is almost always much more accurate than using the time of the simple maximum of TE. Moreover, the value of 95% is sufficiently conservative to assure that the effectiveness of the pellet triggering is not overestimated.

In Table 2, the lag times calculated with the TE have been reported, together with the efficiency of the ELM pacing, calculated as the number of pellets triggering an ELM divided by the total number of pellets. A pellet is considered to have triggered the subsequent ELM if it has reached the plasma within the time lag identified by TE. For completeness, the efficiency of the pellet triggering is reported also for the case of 2 ms lag time, traditionally used in JET. As expected from the numerical tests described in Section 3, Pcc and MI are prohibitively difficult to interpret and provide completely unreliable results.

An important observation, emerging from the application of TE to this type of synchronization experiments, is that the discharges can exhibit a very different behaviour. Therefore the assumption of a single 2 ms time interval, for the causality horizon of ELMs triggering by pellets, is not supported by the present analysis, since the lag times found with TE range between 1.5 and 4.5 ms. The 2 ms interval previously assumed is probably an acceptable estimate on average, but should be particularized for each discharge. Indeed, assuming a fixed 2 ms interval, the actual triggering efficiency can be either overestimated or underestimated depending on the shot. Such a choice can, therefore, be misleading and compromise the interpretation of the actions taken in the experiments, which can be wrongly attributed to the opposite effect on the triggering efficiency than the one they really have. 

## 5. Conclusions

In this paper, some representative examples of the performed systematic numerical tests are reported to show the competitive advantage of transfer entropy with respect to more traditional indicators of causal influence, such as the Pearson correlation coefficient and the mutual information. Particular attention has been devoted to the determination of the causal horizon of a driving force, since this is the case of many delicate synchronization experiments, which have recently attracted a great deal of attention in many disciplines. If causality between different systems includes nonlinear, seasonal, or memory effects, TE always outperforms the other two indicators. The improved results of TE are not marginal, but very substantial. Indeed TE can achieve very accurate results about the time horizon of causal influence for cases when the Pearson correlation coefficient and the mutual information are at a complete loss and do not provide any useful information. In terms of practical applications, the very important examples in magnetic confinement nuclear fusion, the sawteeth pacing with ICRH modulation and the ELM pacing with pellets, have proved the potential of TE in handling complex real time series. The involved nature of the signals and the intricacies of the causal relations render the Pearson correlation coefficient and the mutual information of very limited use in these applications, whereas TE provides very clear results in harmony with other sophisticated indicators and with theoretical considerations [15,16,17]. 

Even if TE clearly outperforms the other indicators in the analysis of the experimental signals investigated, the numerical tests with synthetic spikes have shown a significant problem in detecting the causality direction for this typology of time series. This evidence complements the other limitations of TE recently reported in the literature [18]. Numerical examples have proved that the causal influences can be either underestimated or overestimated by TE, particularly when the relationship between the systems is not dyadic, but polyadic. The complex situations, shown to affect the quality of TE estimates, are relevant not only in general but also for further applications to thermonuclear plasmas. However, the results of TE for the experiments investigated in the present paper seem to be quite robust. The details of the interactions between the driver and the target are not really relevant, given the fact that the experiments are perturbative and the causality directionality is already known. Additionally, the numerical examples provided show that TE can deliver quite accurate results for cases of the same or higher complexity that the experimental signals. 

With regard to future applications, potential phenomena worth investigating with TE are disruptions [19] and LH transition [20,21]. In terms of methodological studies, having assessed the relative merits of TE with respect to traditional indicators, such as the Pearson correlation coefficient and the mutual information, the next step would be to compare its performance with more advanced and recent techniques to assess causality between time series. In addition to advanced versions of the already mentioned Granger causality [22,23], obvious candidates for comparison are cross recurrent plots, cross visibility networks and cross map smoothness. The use of the geodesic distance on Gaussian manifolds to better handle the noise is also to be further pursued [24]. 

## Figures and Tables

**Table 1:** Comparison of the estimated time of the maximum causal influence derived with Pearson correlation coefficient (Pcc), mutual information (MI), and transfer entropy (TE), together with the slowing-down time of the minority (hydrogen) ions. The pulse number identifies the experiments in JET database.

Pulse Number	Regime	Pearson Coefficient (ms)	Mutual Information (ms)	TE: Interval of Maximum Causal Influence (ms)	Slowing-Down Time (ms)
89526	L	24.02 ± 21.44	55.33 ± 3.00	35 ± 1	60 ± 25
89820	L	37.74 ± 38.60	23.59 ± 3.86	55 ± 2	60 ± 25
89822	L	37.74 ± 25.73	96.50 ± 3.34	63 ± 5	50 ± 10
89823	L	15.44 ± 15.01	15.87 ± 2.14	88 ± 2	60 ± 25
89826	L	45.03 ± 25.73	12.87 ± 7.72	45 ± 10	50 ± 10
89999	H	56.80 ± 36.00	48.00 ± 8.00	69 ± 8	60 ± 20
90005	H	72.00 ± 32.00	10.40 ± 4.00	65 ± 9	80 ± 20
90006	H	68.00 ± 24.00	11.20 ± 4.40	75 ± 5	80 ± 20
90007	H	80.00 ± 28.00	14.40 ± 3.20	90 ± 8	100 ± 40

**Table 2:** Percentage of triggering for the lag times calculated with TE and with the usually assumed of 2 ms. The percentages are calculated from the ratio of the number of Edge Localized Modes (ELMs) triggered by pellets, divided by the total number of pellets reaching the plasma for each shot. The pulse number identifies the experiment in JET database.

Pulse	Δt TE (ms)	TE% Triggering	Δt = 2 (ms)	Percentage of Triggering Using 2 ms
82885	1.5	6	2	7
82886	3.8	17	2	6
82887	4.1	23	2	11
82889	4.5	21	2	4
84688	1.3	3	2	9
84690	3.2	21	2	14
84693	3.5	28	2	17
84696	3.5	9	2	2

## References

[1] Bossomaier T., Barnett T., Harré M., Lizier J.T. (2016). An Introduction to Transfer Entropy: Information Flow in Complex Systems.

[2] Schreiber T. (2000). Measuring Information Transfer. Phys. Rev. Lett..

[3] Granger C.W. (1969). Investigating Causal Relations by Econometric Models and Cross-spectral Methods. Econometrica.

[4] Nilsson M., Kleijn W.B. (2007). On the Estimation of Differential Entropy From Data Located on Embedded Manifolds. IEEE Trans. Inf. Theory.

[5] Kruskal M.D., Oberman C.R. (1958). On the Stability of Plasma in Static Equilibrium. Phys. Fluids.

[6] Bussac M.N., Pellat R., Edery D., Soulé J.L. (1975). Internal Kink Modes in Toroidal Plasmas with Circular Cross Sections. Phys. Rev. Lett..

[7] Von Goeler S., Stodiek W., Sauthoff N. (1974). Studies of Internal Disruptions and m = 1 Oscillations in Tokamak Discharges with Soft—X-Ray Tecniques. Phys. Rev. Lett..

[8] Wesson J. (2004). Tokamaks.

[9] Sharapov S.E., Alper D.B., Eriksson L.-G., Fasoli A., Gill R.D., Gondhalekar A., Gormezano C., Heeter R.F., Huysmans G.T.A. (2000). Energetic particle physics in JET. Nucl. Fusion.

[10] Lerche E., Goniche M., Jacquet P., Van Eester D., Bobkov V., Colas L., Giroud I., Monakhov F., Casson F.J., Rimini F. (2016). Rimini Optimization of ICRH for core impurity control in JET-ILW. Nucl. Fusion.

[11] Graves J.P., Lennholm M., Chapman I.T., Lerche E., Reich M., Alper B., Bobkov V., Dumont R., Faustin J.M., Jacquet P. (2014). Sawtooth control in JET with ITER relevant low field side resonance ion cyclotron resonance heating and ITER-like wall. Plasma Phys. Control. Fusion.

[12] Frigione D., Garzotti L., Lennholm M., Alper B., Artaserse G., Bennett P., Giovannozzi E., Eich T., Kocsis G., Lang P.T. (2015). Divertor load footprint of ELMs in pellet triggering and pacing experiments at JET. J. Nucl. Mater..

[13] Garzotti L., Lang P.T., Alonso A., Alper B., Belonohy E., Boboc A., Devaux S., Eich T., Frigione D., Gál K. Investigating pellet ELM triggering physics using the new small size pellet launcher at JET. Proceedings of the 37th EPS Conference on Plasma Physics.

[14] Lerche E., Lennholm M., Carvalho I.S., Dumortier P., Durodie F., Van Eester D., Graves J., Jacquet P., Murari A., JET Contributors (2017). Sawtooth pacing with on-axis ICRH modulation in JET-ILW. Nucl. Fusion.

[15] Murari A., Craciunescu T., Peluso E., Lerche E., Gelfusa M., JET Contributors (2017). On efficiency and interpretation of sawteeth pacing with on-axis ICRH modulation in JET. Nucl. Fusion.

[16] Murari A., Craciunescu T., Peluso E., Gelfusa M., Lungaroni M., Garzotti L., Frigione D., Gaudio P., JET Contributors (2016). How to assess the efficiency of synchronization experiments in tokamaks. Nucl. Fusion.

[17] Murari A., Peluso E., Gelfusa M., Garzotti L., Frigione D., Lungaroni M., Pisano F., Gaudio P. (2016). Application of transfer entropy to causality detection and synchronization experiments in tokamaks. Nucl. Fusion.

[18] James R.G., Barnett N., Crutchfield J.P. (2016). Information flows? A critique of transfer entropies. Phys. Rev. Lett..

[19] Murari A., Vagliasindi G., Arena P., Fortuna L., Barana O., Johnson M., JET-EFDA Contributors (2008). Prototype of an adaptive disruption predictor for JET based on fuzzy logic and regression trees. Nucl. Fusion.

[20] Murari A., Lupelli I., Gelfusa M., Gaudio P. (2013). Non-power law scaling for access to the H-mode in tokamaks via symbolic regression. Nucl. Fusion.

[21] Peluso E., Murari A., Gelfusa M., Gaudio P. (2014). A statistical method for model extraction and model selection applied to the temperature scaling of the L–H transition. Plasma Phys. Control. Fusion.

[22] Yang F., Duan P., Shah S.L., Chen T. (2014). Capturing Connectivity and Causality in Complex Industrial Processes.

[23] Duan P., Yang F., Sirish L.S., Chen T. (2015). Transfer zero-entropy and its application for capturing cause and effect relationship between variables. IEEE Trans. Control Syst. Technol..

[24] Craciunescu T., Murari A. (2016). Geodesic distance on Gaussian manifolds for the robust identification of chaotic systems. Nonlinear Dyn..

